# Supplementary material for: Reprogramming of Amino Acid Metabolism Differs between Community-Acquired Pneumonia and Infection-Associated Exacerbation of Chronic Obstructive Pulmonary Disease
Source: Cells. 2022 Jul 24;11(15):2283. doi: 10.3390/cells11152283 (PMC9330610; doi:10.3390/cells11152283)
Supplement: Supplementary file 1 [file cells-11-02283-s001.zip › cells-1799557-supplementary.pdf]

Supplement

Reprogramming of amino acid metabolism differs between community-acquired pneumonia and infection-associated exacerbation of chronic obstructive pulmonary disease

Haroon Arshad, Anastasios Siokis, Raimo Franke, Aamna Habib, Juan Carlos Lopez Alfonso, Yuliya Poliakova, Eva Lücke, Katina Michaelis, Mark Brönstrup, Michael Meyer-Hermann, Ursula Bilitewski, Jordi Vila, Laurent Abel, Thomas Illig, Jens Schreiber, Frank Pessler

Table of Contents

Figure S1. Mean plasma concentrations of amino acids and amino acid metabolites.

Figure S2. Identification of analytes to be used for subsequent analyses.

Figure S3. Impact of self-reported cardiovascular disease and diabetes on concentrations of Met, ADMA, and SDMA in CAP and COPD.

Table S1. Inclusion and exclusion criteria.

Table S2. List of metabolism indicators.

Table S3. Identification of diagnostic biomarkers by ROC curve analysis.

Table S4. Results of oversampling procedure, modeling the effect of increasing sample size of COPD to sample size of CAP ($n = 29$).

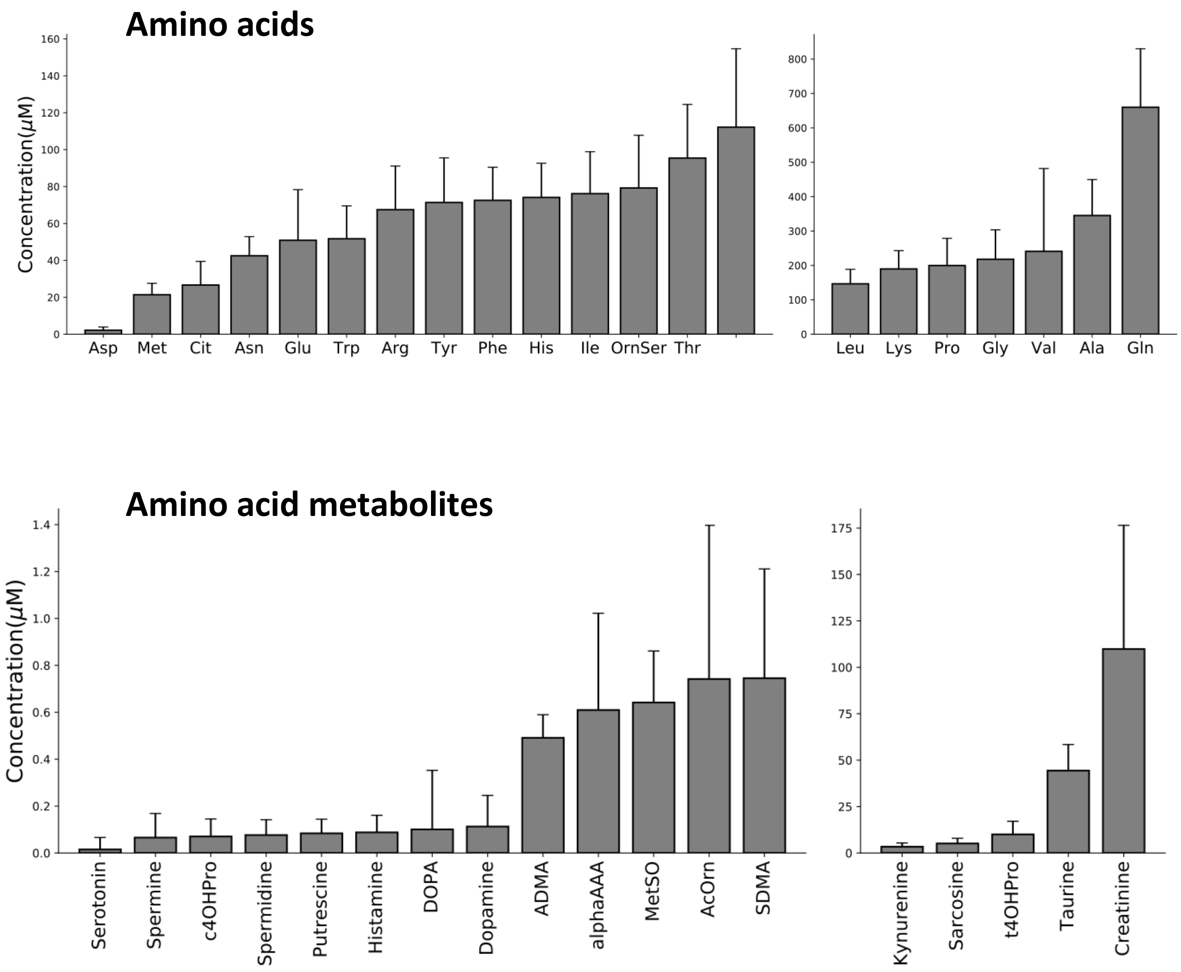

Figure S1. Mean plasma concentrations of amino acids and amino acid metabolites. Values are based on all 21 amino acids and 21 amino acid metabolites detectable with the targeted metabolomic approach used (AbsoluteIDQ™ p180 kit, Biocrates Life Sciences, Innsbruck, Austria). Bars = standard deviation.

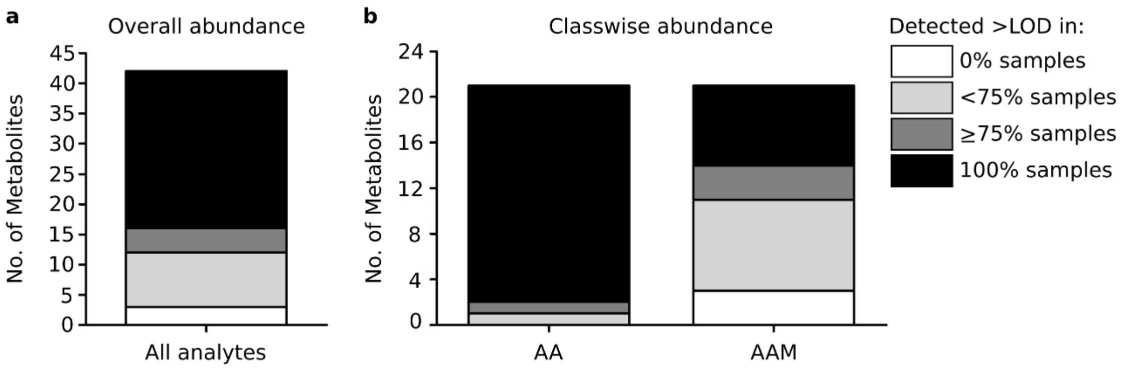

Figure S2. Identification of analytes to be used for subsequent analyses. The percentage of samples in which the analytes were detected >LOD is indicated by the fill darkness. Only analytes detected >LOD in ≥75% of the samples (black and dark grey fill) were included in subsequent analyses. **(A)** Of the 21 potentially detectable amino acids, 20 (95%) were detected >LOD in ≥75% of the samples. (**B**) Of the 21 potentially detectable amino acid metabolites 10 (48%) were detected >LOD in ≥75% of the samples. AA = amino acids; AAM = amino acid metabolites.

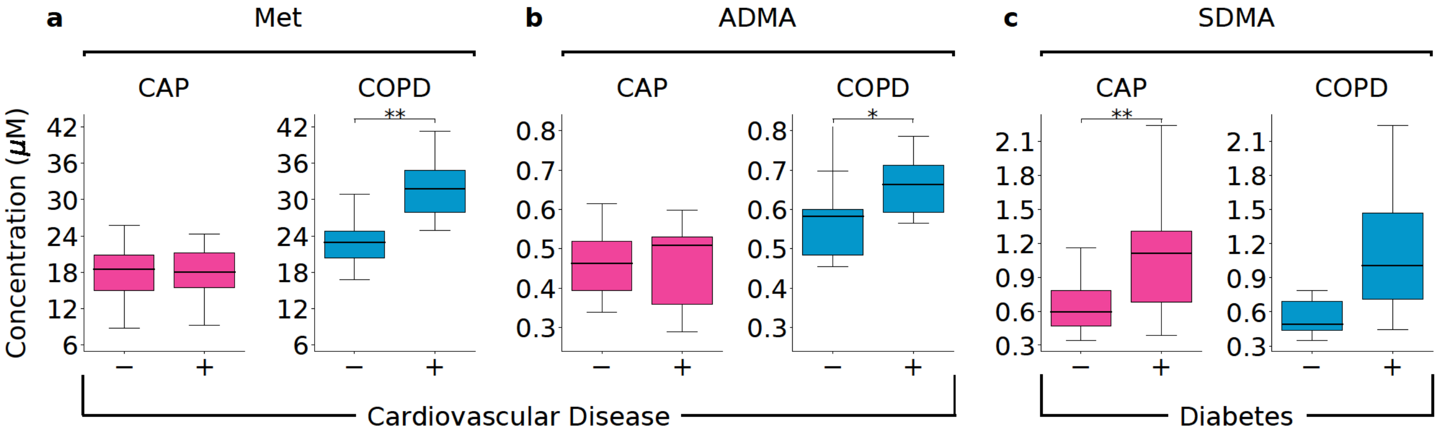

Figure S3. Impact of self-reported cardiovascular disease and diabetes on concentrations of Met, ADMA, and SDMA in CAP and COPD. Data were extracted from the data set used for Figures 1 and 2, but stratified according to self-reported diabetes (CAP) and cardiovascular disease (COPD). (**a**) Met (methionine), (**b**) ADMA (asymmetric dimethylarginine), (**c**) SDMA (symmetric dimethylarginine).

Supplemental Tables

**Table S1.** Inclusion and exclusion criteria	
**Inclusion criteria**	
	**CAP**
	Pulmonary infiltrate on chest x-ray
	At least two of the following four symptoms: cough, purulent sputum production, dyspnea, pleuritic chest pain
	At least two out of fever/hypothermia, hypotension, tachycardia, tachypnea
	At least one out of hypoxemia, evidence of pulmonary consolidation, leukocytosis or leukopenia
	Need for inpatient treatment as judged by the examining physician
	**Infection-triggered COPD exacerbation**
	Documented history of COPD
	Sudden deterioration in respiratory status/lung function
	Symptoms (increase in sputum production, cough) and/or laboratory abnormalities (increased CRP and/or detection of a respiratory pathogen) suggestive of an infectious trigger or coexisting airway infection
	No radiographic evidence of pneumonia
	**Control group**
	No evidence of chronic or acute lung disease
	No evidence of acute or chronic inflammation
**Exclusion criteria**	
	Chemotherapy
	Immunosuppression other than corticosteroids

**Table S2. List of metabolism indicators***			
**Sums (mean concentrations)**		**Description**	**Metabolic significance****
1	BCAA	Sum of branched chain amino acids	Indicator of short-term metabolic control
2	Essential AA	Sum of essential amino acids	Indicator of nutritional status
3	Glucogenic AA	Sum of selected glucogenic amino acids (alanine, glycine, serine)	Indicator of glycolytic vs. gluconeogenic activity
4	Glycolysis	Ala+Gly+Ser	Glycolysis intermediates produced from amino acid metabolism
5	Essential AA	Sum of essential amino acids	Indicator of nutritional status
6	Non-essential AA	Sum of non-essential amino acids	n/s
7	Total AA	Sum of all amino acids	Indicator of nutritional status
	**Ratios involving sums**		
1	Total DMA / Arg	Ratio of dimethylarginine to arginine	Activity of protein arginine methyl transferases (PRMT)
**Ratios of individual amino acids and amino acid metabolites**			
1	Tyr / Phe	Ratio of tyrosine to phenylalanine	Activity of phenylalanine hydroxylase
2	Thr / Ser	Ratio of threonine to serine	KORA Study associates with rs541503 SNP in the PHGDH locus
3	Asn / Gln	Ratio of asparagine to glutamine	Activity of asparagine synthetase
4	Cit / Arg	Ratio of citruline to arginine	Activity of NO synthase
5	Cit / Orn	Ratio of citruline to ornithine	Activity of ornithine carbamoylphosphate transferase
6	DOPA/Tyr	Ratio of DOPA to Tyrosine	Activity of tyrosine hydroxylase enzyme
7	Gln / Asn	Ratio of glutamine to asparagine	Activity of asparagine synthetase
8	Glu/Gln	Ratio of glutamic acid to glutamine	Activity of glutaminase
9	Gly / Arg	Ratio of glycine to arginine	KORA Study associates with rs2216405 polymorphism in the CPS1 locus
10	Gly / Gln	Ratio of glycine to glutamine	KORA Study associates with rs2216405 polymorphism in the CPS1 locus

11	Gly / His	Ratio of glycine to histamine	KORA Study associates with rs2216405 polymorphism in the CPS1 locus
12	Kynurenine / Trp	Ratio of kynurenine to tryptophan	Activity of tryptophan 2, 3-dioxygenase which is the first step in the pathway to niacin. Kynurenine metabolites mediate immunological responses.
13	Met-SO / Met	Ratio of methionine sulphate to methionine	Sulphonation is associated with immune modulation
14	Orn / Arg	Ratio of ornithine to arginine	Activity of arginase
15	Orn / Ser	Ratio of ornithine to serine	KORA Study associates with rs541503 SNP in the PHGDH locus
16	Putrescine / Orn	Ratio of putrescine to ornithine	Activity of ornithine decarboxylase
17	Serotonin / Trp	Ratio of serotonin to tryptophan	Rate of tryptophan degradation to serotonin
18	Spermidine / Putrescine	Ratio of spermidine to putrescine	Activity of spermidine synthase
19	Spermine / Spermidine	Ratio of spermine to spermidine	Activity of spermine synthase
* Adapted from (1).<br>**If indicative of a specific metabolic process.			

Table S3 (pertains to Fig. 4): Identification of diagnostic biomarkers by ROC curve analysis. The symbol * in the Analyte/Indicator column indicates most robust biomarkers as defined by AUC ≥ 0.8, asymptotic p < 0.05, and AUC lower bound 95% CI ≥ 0.5.

**To Fig. 4A (CAP vs. Ctrl)**				
**Rank**	**Analyte**	**Mean AUC [95% CI]**	**Ratio of medians**	**p-value**
1	Trp*	0.96 [0.81, 1.0]	0.64	2.68E-10
2	His*	0.94 [0.81, 1.0]	0.68	1.49E-09
3	Ala*	0.93 [0.77, 1.0]	0.6	3.78E-09
4	Cit*	0.90 [0.67, 1.0]	0.44	3.02E-08
5	Gln*	0.86 [0.64, 1.0]	0.71	7.51E-07
6	Thr*	0.83 [0.60, 1.0]	0.69	3.26E-06
7	t4OHPro*	0.82 [0.58, 1.0]	0.54	7.40E-06
8	Pro*	0.82 [0.62, 0.98]	0.68	1.08E-05
**To Fig. 4B (COPD vs. Ctrl)**				
**Rank**	**Analyte**	**Mean AUC [95% CI]**	**Ratio of medians**	**p-value**
1	ADMA	0.81 [0.43, 1.0]	1.20	5.88E-04
2	Taurine	0.80 [0.33, 1.0]	0.76	1.24E-03
**To Fig. 4C (CAP vs. COPD)**				
**Rank**	**Lipid**	**Mean AUC [95% CI]**	**Ratio of medians**	**p-value**
1	Asn*	0.92 [0.61, 1.0]	0.68	8.58E-06
2	Thr	0.92 [0.61, 1.0]	0.48	8.55E-06
3	Met	0.89 [0.58, 1.0]	0.68	3.98E-05
4	Gln*	0.88 [0.58, 1.0]	0.62	5.59E-05
5	Tyr	0.87 [0.56, 1.0]	0.63	1.33E-04
6	Cit*	0.86 [0.58, 1.0]	0.52	1.26E-04
7	Orn	0.85 [0.5, 1.0]	0.64	1.26E-04
8	Arg*	0.85 [0.5, 1.0]	0.58	1.56E-04
9	Pro	0.83 [0.5, 1.0]	0.67	2.36E-04
10	Lys	0.82 [0.42, 1.0]	0.66	4.73E-04
11	ADMA	0.82 [0.47, 1.0]	0.8	77.62E-04
12	Trp	0.81 [0.33, 1.0]	0.74	1.05E-03
**To Fig. 4D (CAP vs. Ctrl)**				
**Rank**	**Indicator**	**Mean AUC [95% CI]**	**Ratio of medians**	**p-value**
1	Kyn / Trp*	0.94 [0.79, 1.0]	2.28	1.00E-09
2	Non essential AA*	0.93 [0.74, 1.0]	0.71	4.30E-09
3	Tyr / Phe*	0.92 [0.73, 1.0]	0.63	7.35E-09

4	Glucogenic AA*	0.92 [0.71, 1.0]	0.66	8.68E-09
5	Total AA*	0.91 [0.69, 1.0]	0.77	1.11E-08
**To Fig. 4E (COPD vs. Ctrl)**				
**Rank**	**Indicator**	**Mean AUC [95% CI]**	**Ratio of medians**	**p-value**
**1**	Thr / Ser	0.81 [0.48, 1.0]	1.36	7.24E-04
**To Fig. 4F (CAP vs. COPD)**				
**Rank**	**Indicator**	**Mean AUC [95% CI]**	**Ratio of medians**	**p-value**
**1**	Total AA*	0.93 [0.67, 1.0]	0.74	5.92E-06
**2**	Non essential AA*	0.91 [0.70, 1.0]	0.69	1.16E-05
**3**	Thr / Ser*	0.90 [0.56, 1.0]	0.68	3.35E-05
**4**	Glucogenic AA*	0.86 [0.56, 1.0]	0.70	1.33E-04
**5**	Tyr / Phe*	0.85 [0.50, 1.0]	0.71	1.56E-04

**Table S4.** Results of oversampling procedure, modeling the effect of increasing sample size of COPD to sample size of CAP (*n* = 29).				
**Biomarkers for CAP vs. COPD (ROC analysis)**				
**Rank**	**Analyte**	**Mean AUC [95% CI]**	**Ratio of medians**	**p-value**
**1**	Thr	0.94 [0.8, 1.0]	0.47	1.13E-08
**2**	Asn	0.94 [0.8, 1.0]	0.65	1.51E-08
**3**	Tyr	0.89 [0.67, 1.0]	0.62	2.62E-07
**4**	Met	0.89 [0.63, 1.0]	0.67	4.41E-07
**5**	Arg	0.88 [0.67, 1.0]	0.57	7.66E-07
**6**	Gln	0.87 [0.64, 1.0]	0.62	9.46E-07
**7**	Cit	0.86 [0.63, 1.0]	0.51	2.15E-06
**8**	Trp	0.84 [0.5, 1.0]	0.77	9.53E-06
**9**	Pro	0.84 [0.6, 1.0]	0.67	9.18E-06
**10**	Lys	0.83 [0.6, 1.0]	0.61	1.73E-05
**11**	Ala	0.83 [0.57, 1.0]	0.63	1.19E-05
**12**	ADMA	0.82 [0.57, 1.0]	0.8	2.30E-05

Reference

1. Biocrates_Life_Sciences. Factsheet Met*IDQ*™ RatioExplorer 2019. Available from: http://www.biocrates.com/images/FS_RatioExplorer%203.pdf.
